# Disassembly and Mislocalization of AQP4 in Incipient Scar Formation after Experimental Stroke

**DOI:** 10.3390/ijms23031117

**Published:** 2022-01-20

**Authors:** Shervin Banitalebi, Nadia Skauli, Samuel Geiseler, Ole Petter Ottersen, Mahmood Amiry-Moghaddam

**Affiliations:** 1 Division of Anatomy, Department of Molecular Medicine, Institute of Basic Medical Sciences, University of Oslo, 0372 Oslo, Norway; shervinb@studmed.uio.no (S.B.); nadia.skauli@medisin.uio.no (N.S.); ole.petter.ottersen@ki.se (O.P.O.); 2 Cardiovascular Research Group IMB, Department of Medical Biology, Faculty of Health Sciences, UiT—The Arctic University of Norway, 9019 Tromsø, Norway; samuel.geiseler@uit.no; 3 President’s Office, Karolinska Institutet, Nobels väg 6, 171 77 Stockholm, Sweden

**Keywords:** aquaporin-4 (AQP4), AQP4ex, OAP, stroke, ischemia, astrocyte, reactive astrogliosis, glial scar, neuroinflammation

## Abstract

There is an urgent need to better understand the mechanisms involved in scar formation in the brain. It is well known that astrocytes are critically engaged in this process. Here, we analyze incipient scar formation one week after a discrete ischemic insult to the cerebral cortex. We show that the infarct border zone is characterized by pronounced changes in the organization and subcellular localization of the major astrocytic protein AQP4. Specifically, there is a loss of AQP4 from astrocytic endfoot membranes that anchor astrocytes to pericapillary basal laminae and a disassembly of the supramolecular AQP4 complexes that normally abound in these membranes. This disassembly may be mechanistically coupled to a downregulation of the newly discovered AQP4 isoform AQP4ex. AQP4 has adhesive properties and is assumed to facilitate astrocyte mobility by permitting rapid volume changes at the leading edges of migrating astrocytes. Thus, the present findings provide new insight in the molecular basis of incipient scar formation.

## 1. Introduction

Brain injuries—including ischemic events—lead to scar formation in the affected neuropil [1,2]. A mature scar is composed of a central fibrotic component, surrounded by a glial component mainly composed of reactive astrocytes [1,3]. Formation of a glial scar happens in a stepwise fashion starting with upregulation of GFAP which occurs after 2 days in mice followed by astrocytic proliferation and migration towards the injury site [1]. An incipient scar is found after 6–8 days, depending on the severity of injury, and will continue to mature in the following weeks [1]. In cases of cerebral stroke, the glial scar forms around the infarcted area and helps contain the damage. However, the glial scarring is detrimental to neuronal and axonal regeneration [4]. Understanding the mechanisms behind reactive astrogliosis and glial scar formation is integral to therapeutic development and treatment of complications following stroke and other forms of injury to the brain. 

AQP4 is one of the most abundant proteins in brain and is expressed in astrocytes throughout the central nervous system [5]. It has two major isoforms, M1 and M23, based on the alternative translation from methionine residues in position 1 or 23, respectively [6,7]. M23-AQP4 is by far the most prevalent isoform and is known to promote the formation of large supramolecular assemblies called orthogonal arrays of particles (OAPs) [7]. The M1-AQP4 isoform, on the other hand, is found in the periphery of OAPs. It limits the assembly of AQP4 into OAPs and promotes the formation of AQP4 tetramers due to the extended N terminus [6,8,9,10]. Studies indicate that OAPs have adhesive properties and restrict astrocyte mobility, while tetramers promote astrocyte migration [11,12]. Both these processes are of direct relevance to scar formation. Thus, depending on which of the M1 or M23 isoforms that are upregulated, AQP4 may modify or restrict astrocyte mobility and promote migration through their adhesive role and ability to facilitate volume changes in astrocytic processes [13]. Moreover, mice with genetic deletion of AQP4 show reduced glial scar formation and astrocyte migration following stab injury [14].

AQP4ex is a recently discovered isoform of AQP4 created by a translational read-through resulting in a longer C-terminus [15]. This isoform accounts for 10% of the total AQP4 pool in the brain and is necessary for the polarized expression of AQP4 at the perivascular astrocytic membrane domains abutting brain microvessels [16,17]. Little is known about the role and involvement of AQP4ex in pathological conditions.

Here, we demonstrate that AQP4 is radically reorganized in the infarct border zone one week after experimental stroke. A disassembly of supramolecular AQP4 aggregates is associated with a redistribution of AQP4 from perivascular astrocytic membranes to astrocytic processes facing the neuropil. By use of isoform-specific antibodies, we provide evidence for a downregulation of AQP4ex, which is assumed to anchor or stabilize AQP4 molecular assemblies. Our data suggest that reorganization of AQP4 is part of the molecular underpinning of incipient scar formation.

## 2. Results

### 2.1. Mislocalization of AQP4 in the Infarct Border Zone

**Figure 1:**
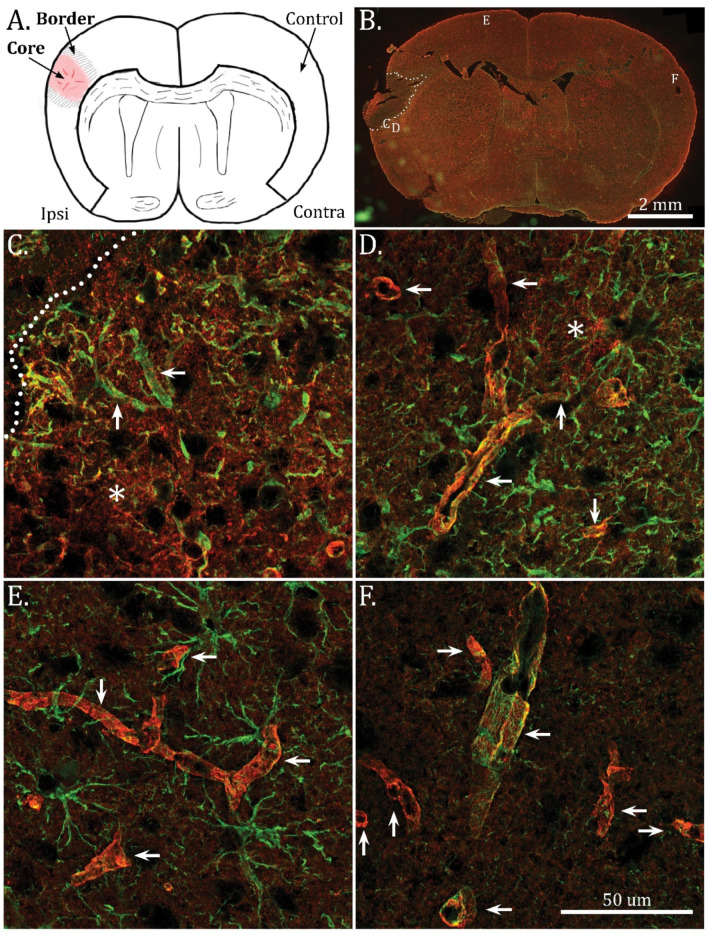
Immunofluorescence shows redistribution of AQP4 from perivascular to non-perivascular domains in the border zone of the infarct. (**A**) Illustration showing the infarct core in the cortex, ipsilateral to the dMCAO. (**B**) Scanned overview of a coronal section with AQP4 (red) and GFAP (green) immunofluorescence co-staining. Dotted lines show the limits of the infarct. Location of the high magnification images shown in (**C**–**F**) is indicated in the scanned overview. (**C**–**F**) Confocal z-projections (2 µm thickness) of areas indicated in B, taken with a 63 × objective. Arrows indicate vessels, asterisk indicate strong AQP4 immunofluorescence staining in neuropil. (**C**) In the border zone closest to the infarct core, GFAP-positive astrocytic processes rectified towards the core (dotted line), are accompanied by diffuse AQP4 immunofluorescence staining throughout the area, with relatively modest immunostaining of the perivascular processes (arrows). (**D**) Moving further away from the core into the peri-infarct area, strong perivascular AQP4 staining (arrows) is accompanied by pronounced neuropil expression (asterisk). Strong GFAP immunofluorescence is present in astrocytic processes. (**E**,**F**). In the ipsilateral cortex remote from the infarct (**E**), and in the contralateral cortex (**F**), AQP4 immunofluorescence is mainly localized around the vessels (arrows). Strong GFAP staining, indicative of astrogliosis is more pronounced in the ipsilateral cortex. Pseudo-colors: red for AQP4 and green for GFAP. Scale Bars: (**A**); 2 mm, (**C**–**F**); 50 µm.

As expected, the distal middle cerebral artery occlusion (dMCAO) led to a limited cortical infarct illustrated in Figure 1A. The infarct core was devoid of GFAP and AQP4 immunofluorescence (Figure 1B, Supplementary Figure S1). On moving away from the infarct core, there was a gradual change in AQP4 immunofluorescence staining intensity and localization. At the infarct edge, AQP4 was diffusely expressed, with no clear perivascular localization (Figure 1C), while more distally in the border zone perivascular AQP4 staining was accompanied by a strong immunofluorescence signal from the neuropil (Figure 1D). In the border zone, scar formation was indicated by intense GFAP staining, and hypertrophic astrocyte processes with rectification towards the infarct core. Astrogliosis extended beyond the infarct into the cortical areas of the ipsilateral hemisphere (Figure 1E). In contrast, AQP4 immunofluorescence staining in cortical areas of the ipsilateral hemisphere remote from the infarct (Figure 1E) was similar to that of the contralateral hemisphere (Figure 1F), indicating that the changes in AQP4 expression pattern were restricted to the infarct and border zone.

**Figure 2:**
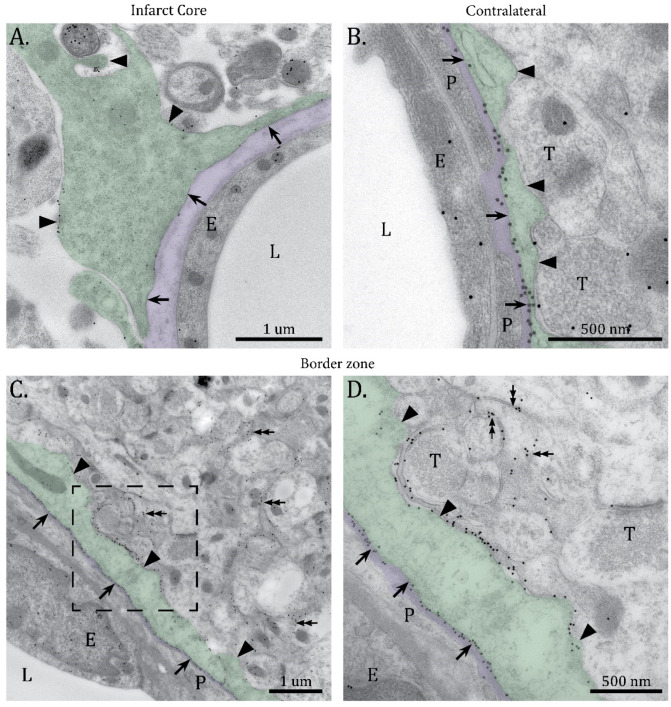
Loss of polarized expression of AQP4 in the border zone. High resolution electron microscopic images showing AQP4 immunogold labeling in the infarct core (**A**), cortex contralateral to the infarct (**B**) and border zone of the infarct (**C**,**D**). (**A**) The perivascular astrocyte endfoot is detached from the subendothelial basal lamina, which seems disintegrated. The astrocyte process is swollen and faint AQP4 immunogold labeling is evenly distributed in the adluminal (arrows) and abluminal (arrowheads) membrane domains. The extracellular space is expanded. (**B**) Highly polarized localization of AQP4 in the adluminal membrane domain (arrows) of a perivascular astrocyte endfoot in the contralateral cortex. Only a few AQP4 immunogold particles are detected in the abluminal membrane domain of the perivascular astrocyte endfoot (arrowheads). (**C**) A hypertrophic perivascular astrocytic endfoot abutting a vessel in the border zone of the infarct. (**D**) High magnification of boxed area in (**C**) shows strong AQP4 immunogold labeling in both the adluminal (arrows) and abluminal (arrowheads) membrane domains of the hypertrophic astrocyte endfoot. Strong AQP4 labeling is also present in the thinner astrocyte processes in the neuropil (double-headed arrows). L; vessel lumen, E; endothelium, P; pericyte, T; axon terminals. Green tint indicates perivascular astrocytic endfeet and purple tint shows the subendothelial basal lamina. Scale bars (**A**,**C**) = 1 µm; (**B**,**D**) = 500 nm.

Immunogold analysis showed faint AQP4 labelling in the core, consistent with the immunofluorescence data. Astrocytic endfeet were swollen and detached from the perivascular basal lamina. The few AQP4 immunogold particles in the core were distributed evenly across all astrocytic membrane domains (Figure 2A), in sharp contrast to the AQP4 labelling remote to the infarct which was concentrated in astrocytic membrane domains facing the subendothelial basal lamina (Figure 2B). In the border zone next to the core, the perivascular endfeet were hypertrophic and a high density of AQP4 immunogold particles was observed in both the adluminal and abluminal membranes of astrocytic endfeet (Figure 2C,D). Strong AQP4 immunogold labeling was also present in astrocytic processes in the neuropil, in line with the immunofluorescence data described above.

### 2.2. Disassembly of AQP4 in Infarct Border Zone

Normally, AQP4 is concentrated in large assemblies named orthogonal arrays of particles (OAPs) after their distinct ultrastructural appearance in freeze fracture specimens [18]. As OAPs are anchored to the adluminal endfoot membrane domains [19], we hypothesized that the loss of AQP4 from these domains in the border zone (Figure 1 and Figure 2) reflects a dissociation of these molecular assemblies.

**Figure 3:**
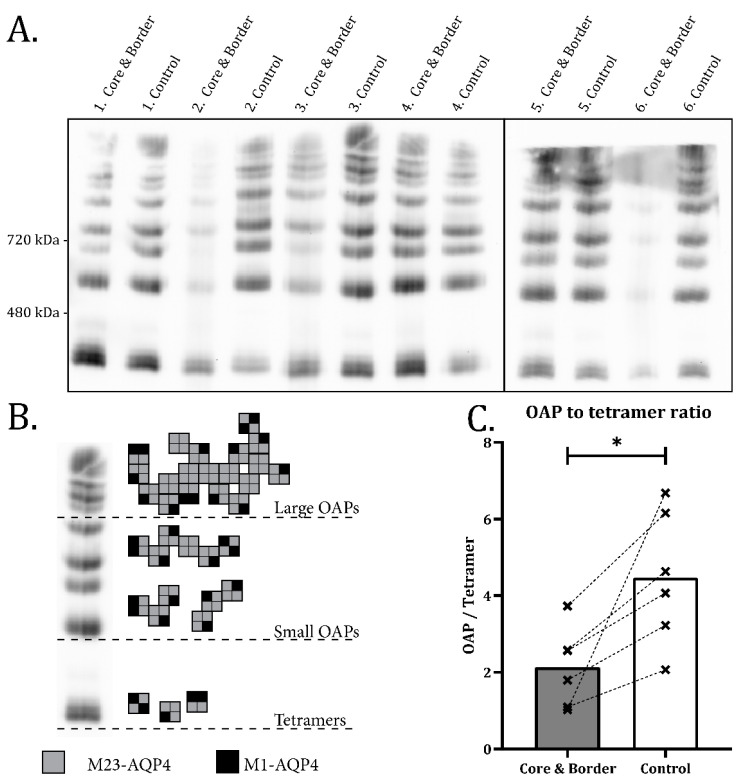
OAP to tetramer ratio is halved in infarct samples revealed by BN-PAGE. (**A**) BN-PAGE of infarct (including both core and immediate border zone) and contralateral control samples immunostained with an anti-AQP4 antibody. (**B**) Illustration showing the separation of tertiary AQP4 structures in BN-PAGE. A tetramer band is followed by bands with higher molecular weight representing OAPs. Large and small OAPs are clearly distinguished in 3–12% gels allowing semiquantitative analysis. (**C**) Individual ratios made by dividing the densitometric values of the small OAP bands by the tetramer band seen in (**B**). Dotted lines connect values from the same animal, asterisk indicates significant difference. Paired Student’s *t*-test (*n* = 6) shows a significant (*p* = 0.020) reduction in OAP/Tetramer ratio in infarct samples compared to contralateral control tissue.

With Blue Native PAGE, all the common tetramer and OAP bands [6] are seen in both infarct and control samples (Figure 3A,B). The lower molecular weight OAP bands properly separated by 3–12% gels were used to calculate the OAP to tetramer ratio. Figure 3C shows a 52% (±40%, *p* = 0.020) reduction in OAP to tetramer ratio in the infarct samples (including core and border) compared with the contralateral control. The higher molecular weight OAPs were not included in the quantitative analysis due to poor separation by 3–12% gels. However, there is no indication that higher OAP bands behaved differently than the ones included in the analysis. No change is seen in the OAP to tetramer ratio in the rest of ipsilateral cortex (Supplementary Figure S2).

### 2.3. Reduction in M1-AQP4 and AQP4ex in Border Zone

The Blue Native PAGE analysis pointed to a disassembly of OAPs in incipient scar formation. We hypothesized that this disassembly could be mechanistically coupled to a relative up-regulation of the M1 isoform of AQP4 that is known to promote formation of tetramers rather than OAPs, or a down-regulation of AQP4ex isoform that is known to stabilize or help anchor OAPs in the astrocytic plasma membrane domains facing the subendothelial basal lamina.

**Figure 4:**
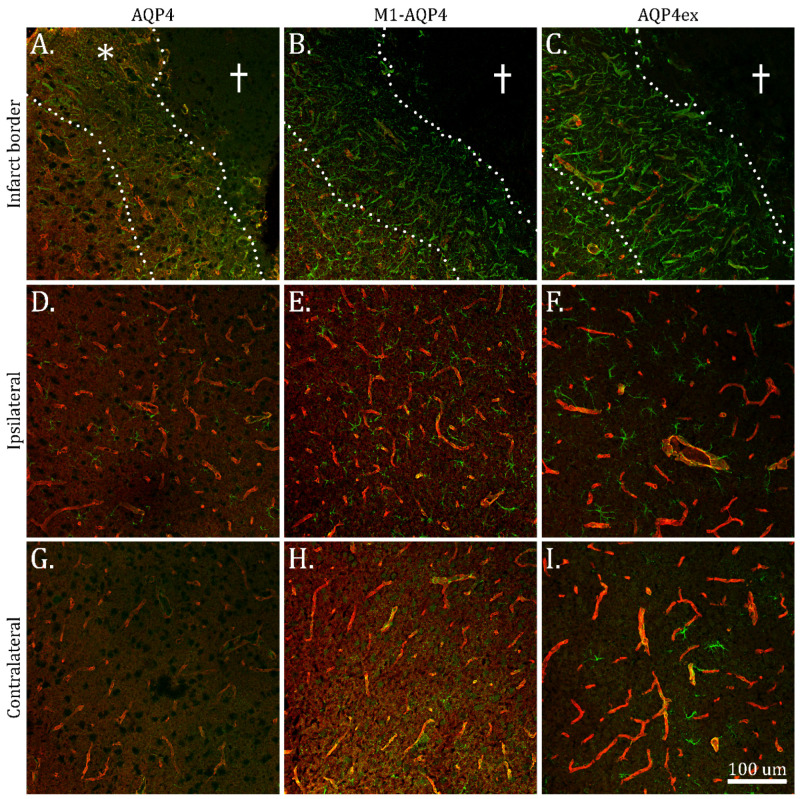
Loss of M1-AQP4 and AQP4ex in the infarct border zone. Confocal immunofluorescence images taken with 20 × objective. (**A**–**C**) Dotted lines indicate extent of incipient glial scar, the cross shows infarct core. (**A**) AQP4 loses its perivascular polarization in the inner half of the glial scar-forming zone. Pronounced AQP4 staining is present in areas not associated with the perivascular processes (asterisk). In the same location, M1-AQP4 (**B**) and AQP4ex (**C**) are much reduced. Moving outwards from the core, there is an increased expression of perivascular AQP4 (total), M1-AQP4 as well as AQP4ex. Normal AQP4 (**D**), M1-AQP4 (**E**) and AQP4ex (**F**) as well as increased GFAP in reactive astrocytes are seen in the ipsilateral cortex. Contralateral cortex (**F**–**I**) shows normal expression of total AQP4 and its isoforms, and a mild degree of astrogliosis judged by GFAP immunostaining. Pseudo-colors: Red: total AQP4 (**A**,**D**,**G**), M1-AQP4 (**B**,**E**,**H**) or AQP4ex (**C**,**F**,**I**), Green: GFAP. Scale bar 100 µm for all images.

To test this hypothesis, we performed an immunofluorescence analysis using antibodies to total AQP4 (which is predominantly composed of M23-AQP4) and isoform-specific antibodies to M1-AQP4 and AQP4ex. Analysis of the border zone (Figure 4A–C) showed that total AQP4 in the areas adjacent to the core is mainly localized in the neuropil with a gradual increase in perivascular staining as one moves further away from the core (Figure 4A). In contrast, the border zone adjacent to the infarct was devoid of M1-AQP4 (Figure 4B) and AQP4ex (Figure 4C) immunofluorescence, with weak perivascular staining. The immunofluorescence for M1-AQP4 and AQP4ex increased upon moving away from the core. The loss of AQP4ex in the border zone adjacent to the infarct core matches the loss of perivascular total AQP4 (Figure 4A). No changes in total AQP4, M1-AQP4 or AQP4ex staining were found beyond the infarct border zone (Figure 4D–I).

**Figure 5:**
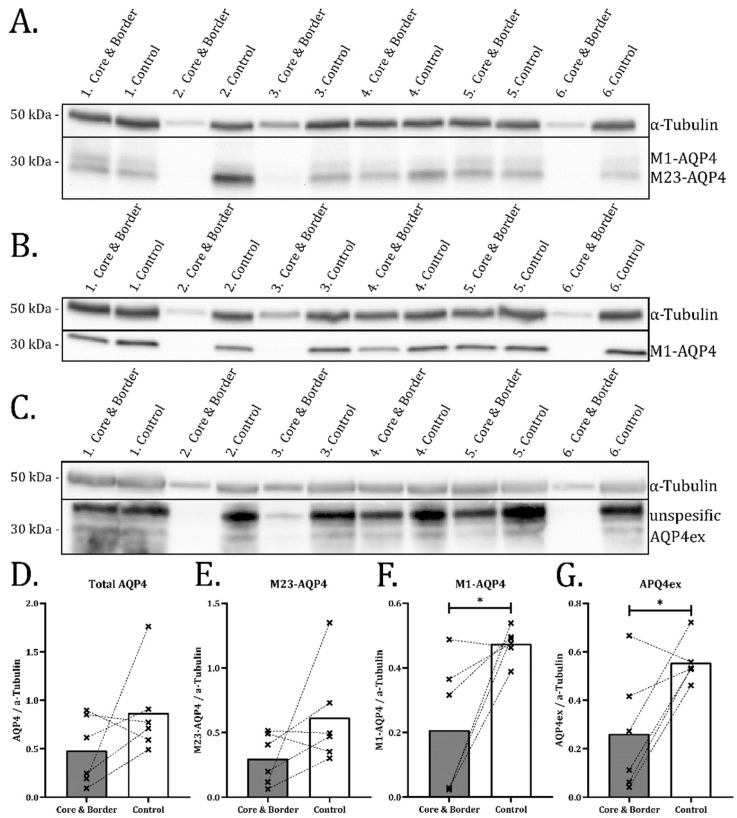
M1-AQP4 and AQP4ex is reduced in infarct samples seen with SDS-PAGE. SDS-PAGE of infarct samples (including core and border zone) and contralateral control. Western blots stained with anti-AQP4 (**A**), anti-M1-AQP4 (**B**) and anti-AQP4ex (**C**). α-Tubulin was used as loading control for all membranes (**A**–**C**) and for normalization in the semiquantitative analysis (**D**–**G**). Normalized densitometric values from (**A**) shown in (**D**,**E**). Values from (**B**) shown in (**F**), and (**C**) in (**G**). (**D**–**G**) dotted lines connect values form the same animal. Paired Student’s *t*-test (*n* = 6) was used for statistical analysis. (**F**,**G**) show significant reductions in M1-AQP4 (*p* = 0.027) and AQP4ex (*p* = 0.028) in infarct tissue (core and border) compared to contralateral control. No statistically significant differences were found for total AQP4 or M23-AQP4 in infarct samples compared to control. Uncut membranes provided in Figure S3. Asterisk indicates significant difference.

To quantify the changes in AQP4 isoform expression, we performed Western blots on protein lysates from tissue samples containing an infarct core and the immediate surrounding tissue including the border zone. The anatomically corresponding region in the contralateral cortex was used as control (Figure 5A–C). Densitometric analysis of the immunoblots showed no significant changes in the levels of total AQP4 (Figure 5D) and M23-AQP4 (Figure 5E) in the samples containing infarct cores and border zones compared to the controls. In the same samples, there was a 56% (±47%, *p* = 0.027) reduction in M1-AQP4 (Figure 5F) and a 53% (±45%, *p* = 0.029) reduction in AQP4ex (Figure 5G) protein level. These findings are consistent with the immunofluorescence data (Figure 4). As the samples also include tissue from the infarct core, which is devoid of AQP4 (as judged by immunohistochemistry), the Western blots indicate an increase in the protein levels of M23-AQP4 in the border zone surrounding the infarct core. This is in line with the immunohistochemical observations (Figure 1, Figure 2 and Figure 4) and suggests a relative isoform-specific downregulation of M1-AQP4 and AQP4ex. No difference in protein expression is seen compared with the rest of the ipsilateral cortex (Supplementary Figure S2).

## 3. Discussion

Effective means to promote repair and reestablish function after stroke and CNS injury require a thorough understanding of the mechanisms underlying scar formation in the brain. It is well known that astrocytes are critically engaged in this process [20]. Astrocytes respond to trauma by changing their morphology and GFAP expression and typically reorganize themselves to migrate towards the site of injury [1]. The border zone of discrete, experimentally induced ischemic infarcts provides a suitable model for analyzing the processes involved in scar formation. During the first week after a stroke, reactive astrocytes undergo characteristic morphological changes leading to the establishment of a glial scar [1]. These changes include rectification of the hypertrophic astroglial processes towards the lesion core. Thus, one week post infarct—the time point chosen for the present study—is held to be optimally suited for investigating the initial steps in the formation of a glial scar [1].

The water channel AQP4 is the most abundant protein in astrocyte plasma membranes and serves a number of roles of putative relevance for scar formation. It has adhesive properties and facilitates astrocyte mobility by permitting rapid volume changes at the leading edges of migrating astrocytes [13,21]. AQP4 is also known to provide an influx route for water during the build-up of brain edema following injury or stroke [22]. This role of AQP4 is of minor relevance in the present study as the experimentally induced infarcts are small and not associated with significant edema.

Early studies [14] suggested that AQP4 is conducive to scar formation following cortical stab injury. Here, we addressed the roles of AQP4 in incipient scar formation using experimental stroke as a model. We hypothesized that in an incipient scar, AQP4 is lost from astrocyte membranes engaged in adhesive functions (i.e., the adluminal membranes of astrocyte endfeet normally attached to the perivascular basal lamina through the AQP4-containing dystrophin complex) and relocalized to other astrocytic membrane domains. This would make the astrocytes more mobile and facilitate their reorientation and migration, given that AQP4 contributes to intercellular adhesion [21]. We also hypothesized that a relocation of AQP4 (if any) would be associated with a disassembly of the supramolecular AQP4 aggregates (OAPs) that normally abound in perivascular astrocytic endfeet.

In line with our hypotheses, we show that AQP4 is mislocalized in the infarct border zone, i.e., at the site of incipient scar formation. The high-resolution immunogold analysis concurred with immunofluorescence data, showing a reduced density of AQP4 from adluminal plasma membrane domains and an accumulation of AQP4 in astrocytic plasma membrane domains in the neuropil. Moreover, we observed swelling and thickening of the perivascular astrocytic endfeet in the infarct core and border zone. This resembles the picture in the α-syntrophin -/- mice which showed thickening of perivascular astrocytic processes [23]. These animals exhibit a pronounced reduction in the amount of AQP4 in adluminal membranes, mimicking the changes seen in the present material. Hence, a likely explanation of the endfoot swelling is that the reduction in the perivascular AQP4 pool interferes with water efflux from the astrocytic processes.

The adluminal membrane of perivascular processes is unique by showing an abundance of protein aggregates called orthogonal arrays of proteins (OAPs). These are supramolecular aggregates of AQP4 as evidenced by the fact that they are absent from mice with targeted deletion of *Aqp4* [24]. Consistent with our hypothesis, we showed that the loss of AQP4 from adluminal membranes is paralleled by a significant reduction in the OAP/tetramer ratio, indicative of a disassembly of supramolecular AQP4 aggregates. The most salient explanation of the mislocalization observed is that the disassembly of OAPs releases AQP4 tetramers that move into non-adluminal membranes through lateral diffusion. An increased number of AQP4 molecules in astrocytic neuropil processes would be likely to facilitate astrocytic reorganization and migration [13,25].

What is the mechanism underlying the relocalization of AQP4 in astrocytic plasma membranes in incipient scar formation? The polarized distribution of AQP4 is perturbed by targeted deletions of α-syntrophin and dystrophin which normally anchor AQP4 to the perivascular basal lamina [19,26]. However, after ischemic stroke, α-syntrophin and dystrophin are retained in perivascular membranes [27], indicating that other mechanisms must be responsible for the loss of perivascular AQP4.

The observed reduction in the level of the AQP4ex isoform provides a possible explanation of the changes observed. Previous studies have shown that genetic deletion of the AQP4ex isoform leads to a reduction in the perivascular pool of AQP4 [17]. In our study, by quantitative immunoblot analysis, we show that AQP4ex expression is decreased by 53% in infarct samples covering the infarct core and border zone. In agreement, immunofluorescence microscopy reveals a complete lack of AQP4ex in the border zone, and a gradual increase in perivascular AQP4ex expression in the surrounding area.

An alternative explanation is that the observed relocalization of AQP4 reflects changes in M1 isoform expression as this AQP4 isoform is known to limit the size of supramolecular aggregates of AQP4 [8]. Our data are not supportive of this explanation. Thus, relative to the total amount of AQP4, M1 appears to decrease rather than increase in specimens that encompass the core as well as the border zone of the cortical infarct.

These findings indicate that there might be undiscovered mechanisms, beyond the ratio between M1 and M23 isoforms of AQP4 that regulate the assembly of the supramolecular AQP4 aggregates.

The tissue samples used for Blue Native and Western blots contained parts of the infarct core as well as the border zone. This notwithstanding, we argue that our conclusions hold as these were guided by our comparative immunogold and immunofluorescence analyses. These analyses allowed us to clearly discriminate between the core, border zone and the cortical zone peripheral to the induced cortical infarct. The strength of this study is the combination of high-resolution immunocytochemistry and blue native gel analysis.

Moreover, our study lacks a functional analysis that directly demonstrates the relevance of the observed findings in the context of scar formation. Here, we draw on previous studies that have provided compelling evidence for a role of AQP4 in facilitating astrocyte migration and mobilization—essential elements in the generation of a scar [13]. The present findings should inspire future studies aimed at disentangling the role of AQP4ex in post-traumatic and post-ischemic scarring and should reinforce our attempts to identify the regulatory mechanisms governing AQP4 assembly and expression.

In conclusion, we have shown that the border zone of discrete cortical infarcts displays a pronounced reorganization and disassembly of AQP4 that may constitute a seminal step in incipient scar formation. The downregulation of the novel AQP4 isoform AQP4ex may hold the key to the observed changes, although alternative mechanisms should be explored in future studies. Targeting the mechanisms underlying AQP4 disassembly and mislocalization could provide a means to modify the reparative response to stroke and brain injury.

## 4. Materials and Methods

### 4.1. Animals, Permanent dMCAO and Dissections

**Figure 6:**
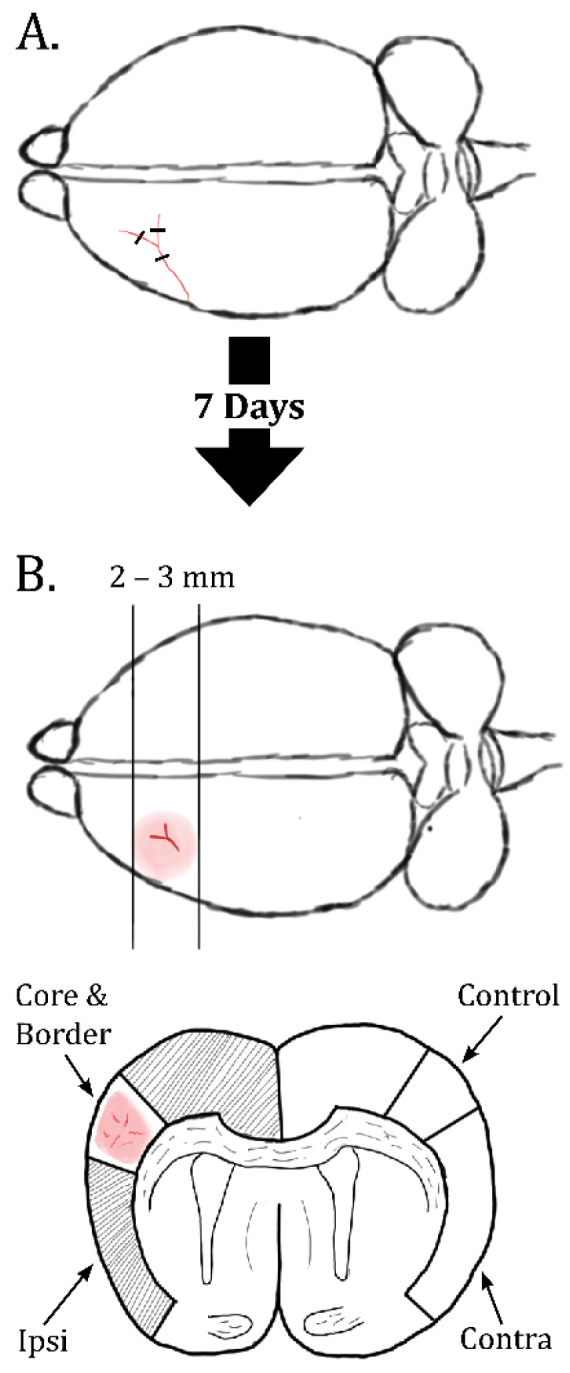
Illustration of dMCAO and dissection. (**A**) The distal parts of the middle cerebral artery are occluded on the surface of the cortex at the bifurcation of the artery. A triple electrocoagulation is used to ensure full occlusion. One week post dMCAO, mice were sacrificed and dissected as shown in (**B**). First a coronal slice was made, encompassing the visible stroke area on the brain surface. Ipsilateral cortex was divided into the visible stroke area (core and border) and rest of ipsilateral cortex (Ipsi), while from contralateral cortex, a control area corresponding to the anatomical location of the infarct area (Control) was separated from the rest of the contralateral cortex (Contra).

Wild-type C57BL/6J mice were acquired from Janvier labs (Le Genest-Saint-Isle, France). Permanent distal middle cerebral artery occlusions (dMCAO) were performed as previously described [28]. In brief, the 38-day-old mice were anesthetized with isoflurane gas (suspended in oxygen) before opening the cranium to reveal the distal middle cerebral artery downstream of the lenticulostriate arteries. A triple electrocoagulation was performed in the bifurcation, as shown in Figure 6A. Buprenorphin 0.1 mg/kg was given in single intraperitoneal injections once a day for four days as post-operative analgesic regiment, and food and water were provided ad libitum. Mice were sacrificed 1 week after stroke induction for sample collection.

For protein extraction, mice were anesthetized with isoflurane and decapitated. The brains were promptly extracted from the skull and dissected (Figure 6B) by coronal sectioning encompassing the visible stroke area. The cortex was divided into the following regions: the Ipsilateral Infarct area (Infarct), the rest of the ipsilateral cortex (Ipsi), the contralateral control (Control) corresponding anatomically to the infarct area, and the rest of the contralateral cortex (Contra). Samples were collected on dry ice and stored at −80 °C until protein extraction.

For immunofluorescence microscopy, mice (*n* = 4) were anesthetized and decapitated. The brains were quickly removed and frozen in isopentane cooled on dry ice before storage at −80 °C until cryosectioning.

For electron microscopy, mice (*n* = 4) were anesthetized with intraperitoneal ZRF (Zolazepam 3.3 mg/mL, Tiletamine 3.3 mg/mL, Xylazine 0.5 mg/mL, Fentanyl 2.6 µg/mL) (1 mL/g) and transcardially perfused with ice-cold 2% dextran in 0.1 M phosphate buffer (PB) for 20 s followed by 4% formaldehyde (FA) and 0.1% glutaraldehyde in 0.1 M PB for 30 min. The brains were removed, post-fixed in the fixation solution overnight, and 1 × 1 × 1 mm samples were dissected from the regions described in Figure 6B.

### 4.2. BN-PAGE

Samples from mouse brains (*n* = 6) were homogenized in a sample buffer (50 mM BisTris, 50 mM NaCl, 6N HCl, 10% *w*/*v* Glycerol, 0.001% Ponceau S, 2.5% *w*/*v* Triton X-100) and protease inhibitor cocktail (Roche, Basel, Switzerland), and the homogenates were centrifuged at 15,000 g for 30 min at 4 °C. The supernatant was transferred to fresh tubes. A Pierce BCA assay (Thermo Fisher, Waltham, MA, USA) was used to determine the protein concentration in the samples.

The protocol used is based on the manual for the BisTris-based NativePAGE gel system by Invitrogen (Waltham, MA, USA) [29]. Prior to electrophoresis, samples were diluted with the sample buffer lacking Triton X-100 and with 5% Coomassie G-250 added equal to ¼ of the Triton X-100 concentration. The final samples with 10 µg protein were used for Blue Native electrophoresis.

Electrophoresis was performed in Xcell SureLock Mini-Cell Electrophoresis System (Thermo Fisher, Waltham, MA, USA) with NativePAGE 3 to 12% 10-well (1.0 mm) Bis-Tris Mini Protein Gels (Thermo Fisher, Cat# BN1001BOX). The upper chamber was filled with dark blue cathode buffer (50 mM BisTris, 50 mM Tricine, 0.4% *w*/*v* Coomassie G-250) and anode buffer (50 mM BisTris, 50 mM Tricine) in the lower chamber. The electrophoresis was run at a constant 6 mA in 4 °C. After 60 min, the upper chamber was emptied and refilled with light blue cathode buffer (50 mM BisTris, 50 mM Tricine, 0.04% *w*/*v* Coomassie G-250). The run was stopped when the dye-front exited the bottom of the gel.

### 4.3. SDS-PAGE on Total Protein Samples

Native protein samples were combined with Laemmli loading buffer (5% β-Mercaptoethanol, 0.02% Bromophenol blue, 30% Glycerol, 10% sodium dodecyl sulphate, 250 mM Tris-Cl) incubated at 37 °C for 15 min to denature the proteins for SDS-PAGE. An amount of 7 µg for total AQP4 and 15 µg for analysis of AQP4 isoforms were used for SDS PAGE.

Electrophoresis was performed in 4–20% Criterion TGX Precast Gels (BioRad, Cat# 5671094) with 1 × Laemmli running buffer (24.8 mM Tris base, 192 mM glycine, 0.1% *w*/*v* sodium dodecyl sulphate) at 120 V for 120 min at room temperature. Precision Plus Protein Dual Color Standard (BioRad, Cat#1610374) was used as a molecular weight marker.

### 4.4. Western Blot and ECL Signal Acquisition

For both BN-PAGE and SDS-PAGE, gels were blotted to PVDF membranes by the Criterion wet transfer system (BioRad). The gel was placed in a sandwich with 0.2 µm PVDF membrane activated in methanol and filter paper on both sides. For BN-PAGE, NuPAGE Transfer Buffer (Invitrogen, Cat# NP0006) was used with Western blotting at 70 V for 60 min. For SDS-PAGE gels 1 × Towbin transfer buffer (24.8 mM Tris base, 192 mM glycine) was used and the blotting run at 100 V for 45 min.

For Blue Native, NativeMark Unstained Protein Standard (Invitrogen) was used and visualized on PVDF membrane by Ponceau S (Sigma-Aldrich (Saint-Louis, MO, USA); Cat# P7170) staining for molecular weight markers.

Membranes were blocked in 5% *w*/*v* Bovine serum albumin in Tris-buffered saline (TBS) (BioRad, Cat# 1706435) for 1 h. The membranes were incubated in primary antibody solution (antibody, 1% BSA, 1 × TBS) containing the relevant primary antibody (Table 1) overnight at 4 °C before washing with 1% Tween-20 in TBS (TBST) and incubated with horseradish peroxidase-bound secondary antibodies (ECL anti rabbit, GE Healthcare (Chicago, IL, USA)). Finally, the membranes were incubated with Super Signal West Pico Chemiluminescent ECL Substrate (Thermo Fisher, Cat# 34580) for 5 min and developed using ChemiDoc MP Imaging System (BioRad).

Images were exported using Image Lab (version 6.0.1, BioRad) and analyzed with Image Studio Lite version 5.2 (LI-COR Biosciences, Lincoln, NE, USA). Statistics were performed with STATA 17 (Statacorp, College Station, TX, USA) using the paired Student’s *t*-test. *p*-values of <0.05 were considered statistically significant. Values are presented as the percent difference of ±1.96 SD. For all SDS PAGE quantifications, α-tubulin was used as an internal control.

### 4.5. Immunogold Transmission Electron Microscopy 

Tissue blocks were subject to freeze substitution as previously described [30]. Blocks were cryo-protected in glycerol before rapidly being frozen in liquid propane at −170 °C. The blocks were immersed in anhydrous methanol with 1.5% uranyl acetate at 90 °C, followed by infiltration with Lowicryl HM20 Resin (Lowy, Waldkraiburg, Germany) at −30 °C and polymerized by ultraviolet light. Ultrathin sections (90–100 nm) were cut and placed on 300 mesh Ni-grids.

Immunogold staining was performed as described [31]. Grids were incubated with TBST (Tris-buffered saline containing 2% Triton X-100) three times for 10 min before incubation with TBST containing 50 mM glycerol for 10 min and then blocked with TBST containing 2% human serum albumin for 10 min. The grids were incubated with a primary antibody (Table 1) in a humid chamber overnight and washed in TBST before incubation with 15 nm gold-conjugated secondary antibody (Table 1) for 90 min. Contrasting was conducted with incubation in 1% uranyl acetate and 0.3% lead citrate for 90 s each with ample distilled H_2_O washes between.

Microscopy and imaging were performed with a Tecnai 12 (FEI Company, Eindhoven, The Netherlands) transmission electron microscope at 80 kV high tension. Colored tints were added with Adobe Illustrator version 16.0 (Adobe systems, San Jose, CA, USA) to highlight areas of interest.

### 4.6. Immunofluorescence Laser Scanning Microscopy

Fresh frozen brains were embedded in OCT and cut with freeze microtome to sections with a thickness of 12 µm. Cut sections were placed on glass slides and incubated with 2% PFA in phosphate-buffered saline (PBS). The PFA was rinsed off 3 times in 5 min incubations of PBS before incubating in blocking solution (2% donkey serum, 0.2% bovine serum albumin and 0.005% Triton X-100 in PBS) for 1 h, then in PBS three times in 5 min before adding primary antibodies (Table 1) and incubating in a humid chamber overnight. The following day, primary antibody solution was washed off and the sections were incubated in secondary antibody (Table 1) for 30 min, before changing to secondary antibody solution containing DyLight 649 (tomato) lectin stain (Vector labs (Burlingame, CA, USA), Cat# DL-1178-1) for another 30 min. The sections were rinsed in PBS and incubated with Hoechst nuclear stain (Invitrogen, H3569) 1:5000 dilution for 5 min before washing with distilled water. The sections were mounted with Prolong Glass Antifade Mountant (Invitrogen, Cat# P36984) and high-precision cover glasses (170 ± 5 µm).

High-resolution images of histological sections were acquired using an automated Axio Scan Z1 slide scanner system (Carl Zeiss Microscopy, Munich, Germany). Confocal images were acquired with an Elyra LSM 710 microscope (Carl Zeiss Microscopy) using 63 × oil-immersed objective. Images were inspected and exported with ZEN blue 3.2 (Carl Zeiss Microscopy).

## Figures and Tables

**Table 1:** Overview of antibodies used.

Method	Primary Antibody	Secondary Antibody
Western Blot (SDS and BN-PAGE)	Anti-AQP4 (rabbit), Sigma Cat# A5971, RRID: AB_258270, 1:5000	ECL anti-rabbit (donkey), GE Healthcare Cat# NA934, RRID: AB_772206, 1:25,000 dilution
	Anti-M1-AQP4 (rabbit), A. Frigeri ^1^, 1:2000	ECL anti-rabbit (donkey), GE Healthcare Cat# NA934, RRID: AB_772206, 1:25,000 dilution
	Anti-AQP4ex (rabbit), A. Frigeri ^1^, 1:2000	ECL anti-rabbit (donkey), GE Healthcare Cat# NA934, RRID: AB_772206, 1:25,000 dilution
	Anti-α-tubulin (rabbit), Abcam Cat# ab4074, RRID: AB_2288001, 1:5000 dilution	ECL anti-rabbit (donkey), GE Healthcare Cat# NA934, RRID: AB_772206, 1:25,000 dilution
Immunofluorescence LSM	Anti-AQP4 (rabbit), Sigma Cat# A5971, RRID: AB_258270, 1:1200	Anti-rabbit Cy3 (donkey), Jackson ImmunoResearch Labs Cat# 711-165-152, RRID: AB_2307443, 1:250 dilution
	Anti-M1-AQP4 (rabbit), A. Frigeri ^1^, 1:2000 dilution	Anti-rabbit Cy3 (donkey), Jackson ImmunoResearch Labs Cat# 711-165-152, RRID: AB_2307443, 1:250 dilution
	Anti-AQP4ex (rabbit), A. Frigeri ^1^, 1:2000 dilution	Anti-rabbit Cy3 (donkey), Jackson ImmunoResearch Labs Cat# 711-165-152, RRID: AB_2307443, 1:250 dilution
	Anti-GFAP (chicken), BioLegend Cat# 829401, RRID:AB_2564929, 1:400 dilution	Anti-chicken Cy2 (donkey), Jackson ImmunoResearch Labs Cat# 703-225-155, RRID: AB_2340370, 1:250 dilution
Immunogold TEM	Anti-AQP4 (rabbit), Sigma Cat# A5971, RRID: AB_258270, 1:400 dilution	Anti-rabbit (Goat) 15nm gold conjugated, Abcam Cat# ab27236, RRID: AB_954457, 1:20 dilution

^1^ Custom-made antibodies validated in [10,15]. Validation of antibodies in SDS PAGE with AQP4 knock out tissue seen in Figure S2.

## Supplementary Materials

The following supporting information can be downloaded at: https://www.mdpi.com/article/10.3390/ijms23031117/s1.
