# Supplementary material for: Disassembly and Mislocalization of AQP4 in Incipient Scar Formation after Experimental Stroke
Source: Int J Mol Sci. 2022 Jan 20;23(3):1117. doi: 10.3390/ijms23031117 (PMC8835637; doi:10.3390/ijms23031117)
Supplement: Supplementary file 1 [file ijms-23-01117-s001.zip › ijms-1546334-supplementary.pdf]

Supplementary Figures

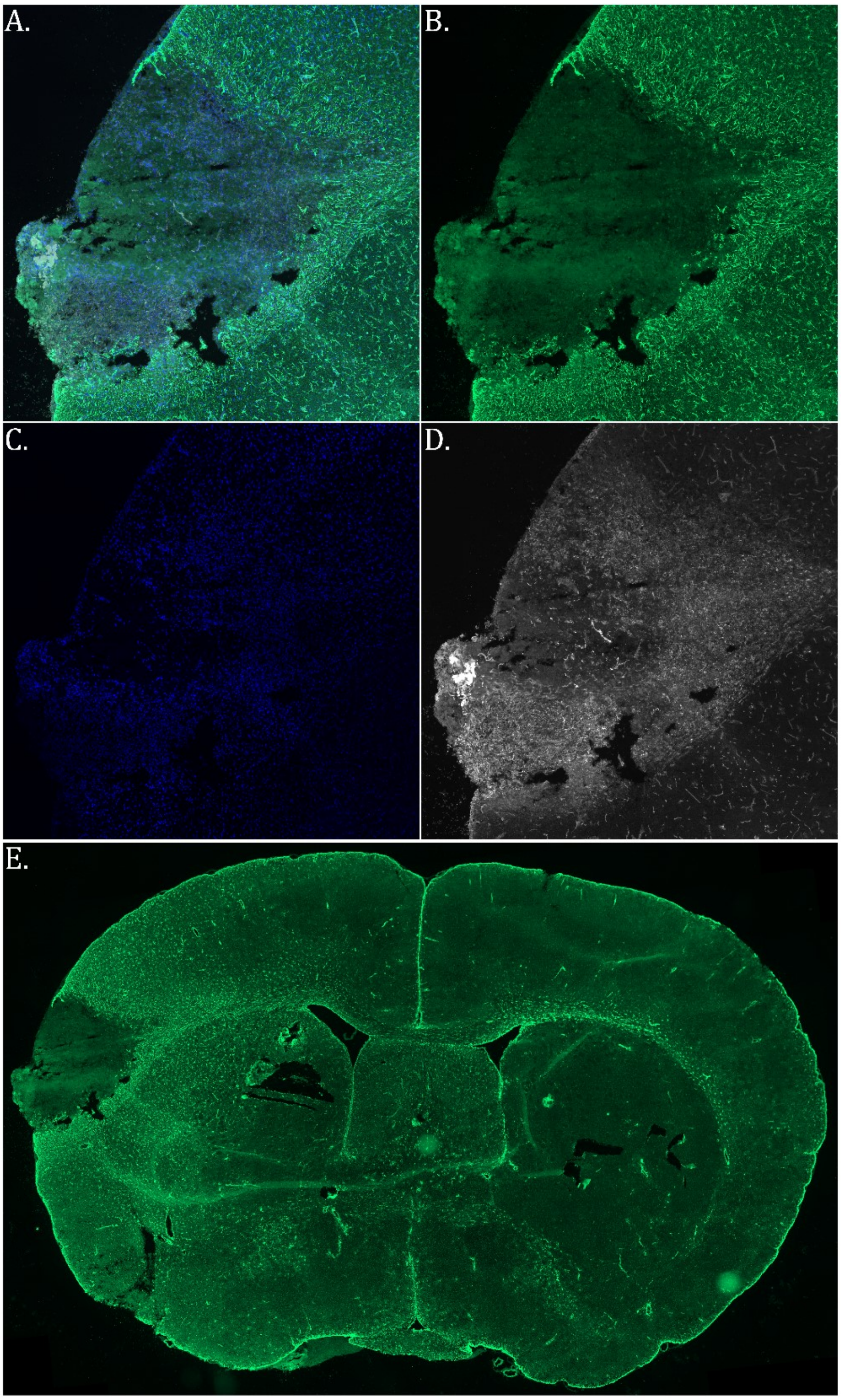

Figure S1. Immunofluorescence of infarct, glial scar and astrogliosis. Scanned immunofluorescent section showing stroke area. Blue shows nuclear stain, green for anti-GFAP and white for lectin stain. (A) The infarct core is devoid of GFAP staining and populated with lectin-positive cells, indicating infiltration of immune cells. (B)Note strong GFAP staining in infarct border, indicating incipient glial scar formation. (C) Shows nuclear stain, and (D) Shows lectin which stains both immune cells and endothelial cells (blood vessels).

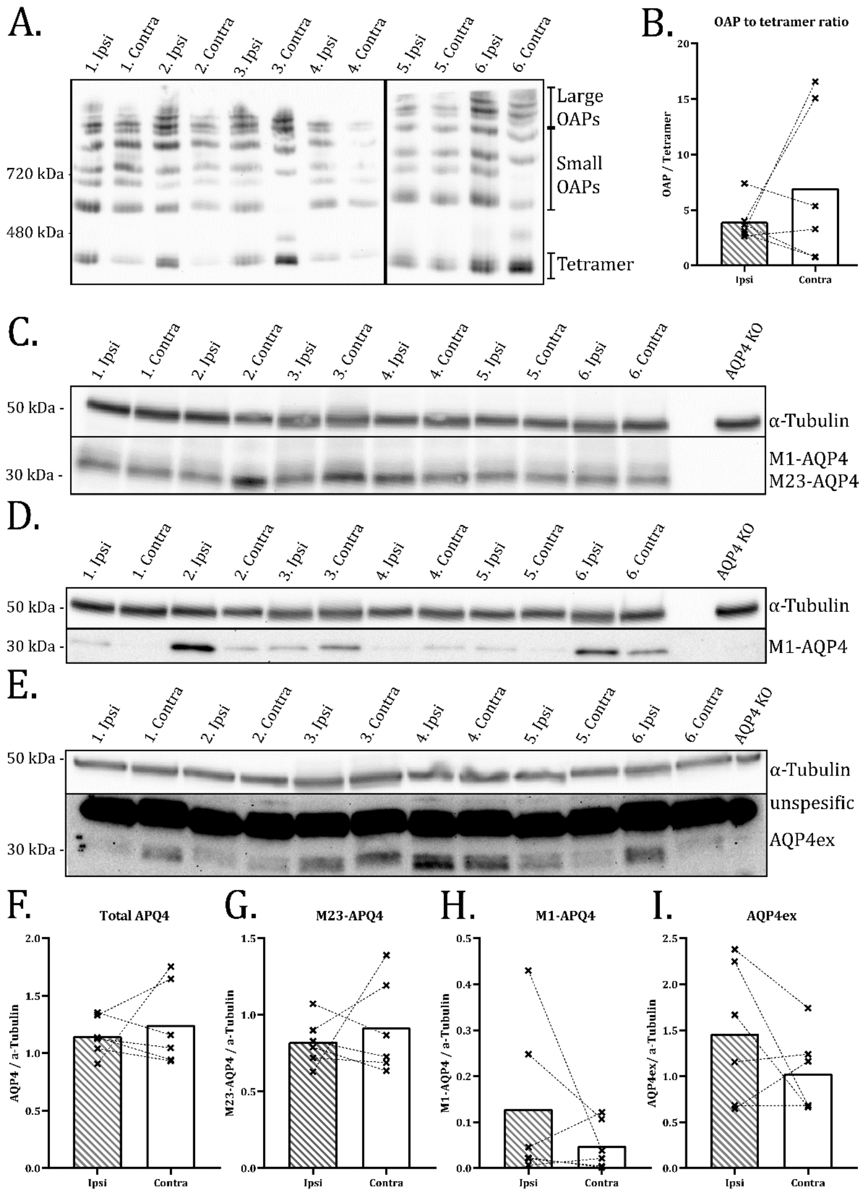

Figure S2. No change in OAPs or isoform expression in ipsilateral cortex
(A) Blue Native PAGE of ipsilateral and contralateral cortex stained with anti-AQP4 showing the distribution of AQP4 in its tertiary structures. (B) Showing ratios of densitometry values of small OAPs divided by tetramer band. Dotted lines connect values form the same animal. Two sample T-test (n=6) shows no significant difference. (C-E) show SDS PAGE of the same samples immunostained with antibodies recognizing total AQP4 (C), M1-AQP4 (D), and AQP4ex (E). (F-I) show normalized densitometric values from (C-D). Dotted lines connect values from the same animal. Paired two sample t-Tests (n=6) show no significant differences between the values from Ipsi and Contra samples. Uncut membranes provided in figure S3.

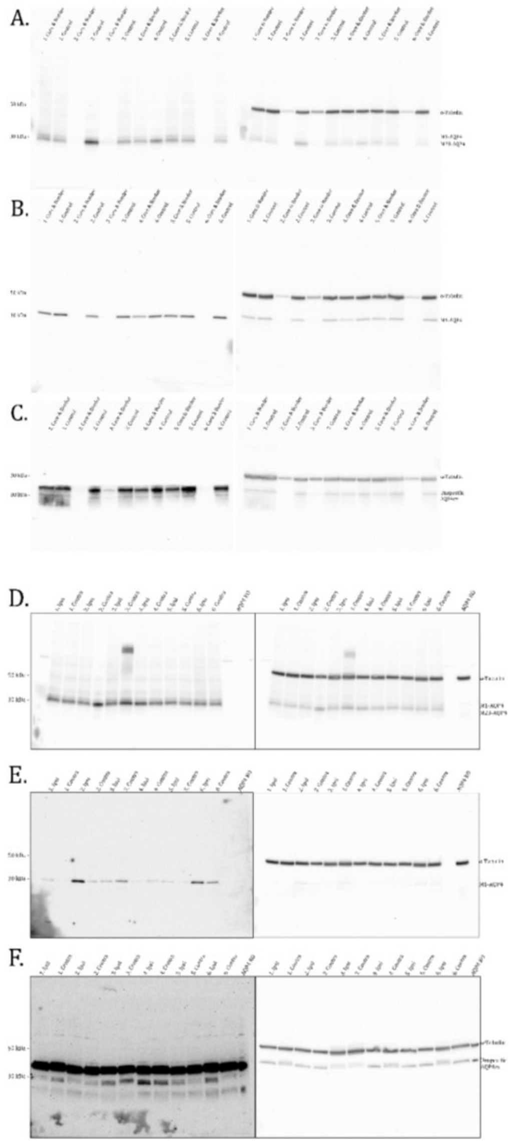

Figure S3. Full SDS PAGE western blotting membranes.

(A-C) shows western membranes for figure 4. (D-E) Shows membranes for Figure S2. Membranes on the left were developed first visualizing AQP4, M1-AQP4 or AQP4ex. Figure on the right shows the same membrane after incubation with anti-aplha-tubulin.
